# Internal Cylindrical Grinding Process of INCONEL^®^ Alloy 600 Using Grinding Wheels with Sol–Gel Alumina and a Synthetic Organosilicon Polymer-Based Impregnate

**DOI:** 10.3390/mi11020115

**Published:** 2020-01-21

**Authors:** Wojciech Kapłonek, Krzysztof Nadolny, Krzysztof Rokosz, Jocelyne Marciano, Mozammel Mia, Danil Yurievich Pimenov, Olga Kulik, Munish Kumar Gupta

**Affiliations:** 1 Department of Production Engineering, Faculty of Mechanical Engineering, Koszalin University of Technology, 75-620 Koszalin, Poland; wojciech.kaplonek@tu.koszalin.pl; 2 Department of Engineering and Informatics Systems, Koszalin University of Technology, 75-620 Koszalin, Poland; krzysztof.rokosz@tu.koszalin.pl; 3 Horiba France S.A.S, Avenue de la Vauve-Passage Jobin Yvon, CS 45002-91120 Palaiseau, France; jocelyne.marciano@horiba.com; 4 Department of Mechanical Engineering, Imperial College London, Exhibition Rd., London SW7 2AZ, UK; m.mia19@imperial.ac.uk; 5 Department of Automated Mechanical Engineering, South Ural State University, 454080 Chelyabinsk, Russia; danil_u@rambler.ru; 6 Department of Mechanical Engineering, Volzhsky Polytechnic Institute (Branch) of Volgograd State Technical University, 404121 Volzhsky, Russia; nio@visteh.net; 7 Key Laboratory of High Efficiency and Clean Mechanical Manufacture, Ministry of Education, School of Mechanical Engineering, Shandong University, Jinan 250061, China; munishguptanit@gmail.com

**Keywords:** impregnation process, silicone, abrasive tools, internal cylindrical grinding, hard-to-cut materials, surface measurements and analysis

## Abstract

The development of modern jet engines would not be possible without dynamically developed nickel–chromium-based superalloys, such as INCONEL^®^ The effective abrasive machining of above materials brings with it many problems and challenges, such as intensive clogging of the grinding wheel active surface (GWAS). This extremely unfavorable effect causes a reduction in the cutting ability of the abrasive tool as well as increase to grinding forces and friction in the whole process. The authors of this work demonstrate that introduction of a synthetic organosilicon polymer-based impregnating substance to the GWAS can significantly improve the effects of carrying out the abrasive process of hard-to-cut materials. Experimental studies were carried out on a set of a silicon-treated small-sized sol–gel alumina 1-35×10×10-SG/F46G10VTO grinding wheels. The set contained abrasive tools after the internal cylindrical grinding process of INCONEL^®^ alloy 600 rings and reference abrasive tools. The condition of the GWAS after the impregnation process was studied, including imaging and measurements of its microgeometry using confocal laser scanning microscopy (CLSM), microanalysis of its elemental distribution using energy dispersive X-ray fluorescence (EDXRF), and the influence of impregnation process on the grinding temperature using infrared thermography (IRT). The obtained results confirmed the correctness of introduction of the impregnating substance into the grinding wheel structure, and it was possible to obtain an abrasive tool with a recommended characteristic. The main favorable features of treated grinding wheel concerning the reduction of adhesion between the GWAS and grinding process products (limitation of the clogging phenomenon) as well as reduction of friction in the grinding process, which has a positive effect on the thermal conditions in the grinding zone.

## 1. Introduction

Among the many advanced materials used in today’s key branches of the aeronautical industry, the hard-to-cut austenitic nickel–chromium-based superalloys, described in detail by Reed et al. [1] and Geddes et al. [2], play the most important role. From the wide group of such materials the INCONEL^®^ family superalloys are significant in terms of the varieties produced, which are characterized by different chemical composition, physical, mechanical, and thermal properties as well as wide range of applications, which was presented by Sharma et al. [3], and Pashmforoush and Bagherinia et al. [4].

One of a typical INCONEL^®^ family superalloy is INCONEL^®^ alloy 600—a standard engineering material for those applications, in which excellent mechanical properties (high strength, good workability), high oxidation and corrosion resistance (including dry Cl_2_ and HCl gasses) as well as high temperature to above 1095 °C (2000 °F) are required, as presented by Ezugwu et al. [5]. 

To obtain the required geometrical parameters of the surface texture of the above elements, they are often precisely machined by the use of modern varieties of grinding processes, well characterized by Denkena and Fischer et al. [6] and Dai et al. [7]. Regardless of the technological process used, the inevitable effect of reducing its effectiveness is intensive clogging of the abrasive tool by ductile chips, which arise as a result of the temperature of sticking the chips of workpiece material to the abrasive grains. Intensive clogging causes a reduction in the cutting ability of the grinding wheel active surface (GWAS), increase to grinding forces, and friction share in the whole process. This is a strongly unfavorable effect which, in industrial practice, tries to reduce considerably by using various types of solutions tailored to the specificity of the given technological process. One of them is the intentional introduction of chemical interactions aimed at lowering the temperature in the grinding zone (GZ) and preventing the adhesion of chips to the workpiece surface and the grinding wheel. This effect can be achieved by, e.g., impregnating abrasive tools, consisting the direct introduction to their active surface or to the entire volume (intergranular space) of additional active substances (impregnate substances) affecting the grinding conditions, as described by Marinescu et al. [8] and Chirkov et al. [9]. 

In the abrasive processes, many types of anti-adhesive substances are used. Historically, one of the first was sulfur, testing as impregnate since the beginning of the 20th century. The method of its introduction on the GWAS was patented by M.L. Harmann in 1927 [10]. Over the next decades, testing and improving also other types of a non-toxic substances based on:

- non-metallic elements and their derivatives (e.g., sulfur, graphite, amorphous carbon);
- metallic elements (e.g., cooper, bismuth alloy);
- organic chemical compound (e.g., wax, paraffin);
- inorganic chemical compound (e.g., aluminum oxide, hexagonal boron nitride);
- solids nanoparticles (e.g., molybdenum disulfide, graphene, diamond);
- organosilicon compounds (e.g., silicone);
- polymer compounds (e.g., epoxy resin).

The variety of substances representing the above groups is relatively wide. The list of selected of them (included reference to patent or paper with the year of its publication) is given in Table 1.

Searching for new impregnating substances, scientists drew attention to multi-molecular organosilicon compounds (organopolysiloxanes)—the silicones. In these synthetic polymer materials, the siloxane skeleton is the main component of their structure. The skeleton is composed of silicon atoms connected by oxygen atoms (in the same way as in inorganic silicates). In addition, various organic groups are attached to the siloxane skeleton through silicon–carbon bonds. To give some silicones special properties, various additives are used, e.g., fillers, emulsifiers, solvents, and water. There are many varieties of silicones that differ from each other in their properties and methods of preparation. These varieties include, above all, silicone oils, emulsions, pastes, greases, natural rubbers, and resins. The high thermal stability of silicones is due to the presence of strong Si–C bonds and Si–O bonds, which have a partially ionic character. The Si–C binding is stable up to 500 °C (932 °F), but only in an anaerobic atmosphere. The resistance of this bond to the oxidation process is much lower, hence the heat resistance of silicones in the presence of oxygen for siloxanes is about 200 °C (392 °F) as reported by Stevents et al. [10]. 

The demand for various silicone polymers is constantly increasing. This is due to their unique properties compared to conventional organic polymers and because a small amount of silicone polymer gives positive results. The most important beneficial features of silicones, are:

- thermal and oxidation resistance over a wide temperature range;
- resistance to weathering, ultraviolet (UV) radiation;
- chemical resistance (except for strong acids/alkalis and certain organic solvents);
- poor electrical conductivity;
- low free surface energy and surface tension;
- small variation of physical properties over a wide temperature range.

**Figure 1:**
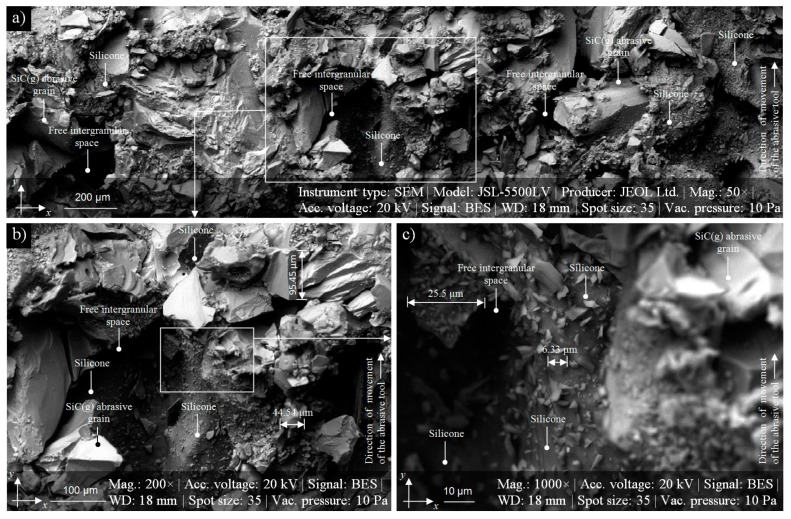
Collection of SEM-micrographs of silicone-treated GWAS 1-35×20×1099C46K8V before the abrasive machining process; obtained using a scanning electron microscope JEOL JSM-5500LV: SEM micrograph (area size: 2980.76 × 2236.53 μm, magnification: 50×) presenting a fragment of vast panorama of the GWAS (**a**); SEM micrograph (area size: 662.39 × 496.58 μm, magnification: 200×) extracted from (**a**) with visible silicon-treated areas of the GWAS (**b**); SEM micrograph (area size: 129.16 × 96.83 μm, magnification: 1000×) extracted from (**b**)—close-up of one of the silicon-treated area of the GWAS (**c**). Note: Some dimension of the abrasive grains was given in (**b**),(**c**).

The above properties contributed to the choice of silicone as an impregnate, which became a very promising substance introduced into the structure of the GWAS to reduce the chip adhesion. In Figure 1, an example of introducing silicone into the structure of the small-sized silicon carbide (green) 1-35×20×1099C46K8V grinding wheel with ceramic bond, is presented. 

The aim of this work is to determine the possibility of relatively easy to use and not expensive modification of the cutting properties of abrasive tools intended for grinding of hard-to-cut materials. The paper considers the problem of introducing the active substance in the form of an impregnate into the abrasive wheel volume as well as demonstrating the potentially beneficial effect of synthetic organosilicon polymer-based impregnate in the internal cylindrical grinding process of INCONEL^®^ alloy 600 carried out using grinding wheels with sol–gel alumina abrasive grains.

In terms of applicability as an impregnating substance, the silicone is interesting. This synthetic organosilicon polymer has already been the subject of the authors’ experimental studies, and its preliminary results were presented by Kapłonek et al. [52] and Nadolny et al. [53]. The authors decided to divide it into two main parts. The first part, covering Section 3, is concerned with selection of abrasive tool and workpiece material, course and effects of the impregnation process and its verification (CLSM, EDXRF), whereas the second part, covering Section 4, is focused on using of the silicone-treated abrasive tool in internal cylindrical grinding process of INCONEL^®^ along with its surface microgeometry condition verification (CLSM) and analysis of the influence of impregnation on the abrasive process temperature (IRT). In the final part of the work, covering Section 5, a summary of all obtained results with their detailed interpretation is presented.

## 2. Materials and Methods

### 2.1. Characteristics of the Abrasive Tools

For experimental studies, a set of ten grinding wheels with a technical designation of 1-35×10× 10-SG/F46G10VTO was prepared. The abrasive tools were produced of sintered microcrystalline alumina abrasive grains and a glass-crystalline ceramic bond, which provided a very open structure, facilitating for a quick penetration of the impregnating substance into the grinding wheel body. General characteristics of the grinding wheels are divided into two groups—silicone-treated grinding wheels (STGW) and a non-impregnated reference grinding wheels (RGW), as presented in Table 2.

### 2.2. Characteristics of the Workpiece Material

For experimental studies, a set of ten samples in a form of rings (internal diameter: *d_w_* = 45 mm, width: *b_w_* = 20 mm) was prepared. As a workpiece material, the hard-to-cut austenitic nickel–chromium-based superalloys—INCONEL^®^ alloy 600 was used. The general characteristics of such material is given in Table 3.

## 3. Impregnation Process of the Abrasive Tools and Its Verification

### 3.1. Impregnation Process of the Abrasive Tools by Colloidal Silicone 

The small-sized sol–gel alumina 1-35×10×10-SG/F46G10VTO grinding wheels were treated with a colloidal synthetic organosilicon polymer (silicone). The impregnating substance became self-acting cross-linked (cured) under the reduce pressure conditions. Additionally, the crosslinking agent (methyltriacetoxysilane) was used which, under the influence of moisture, readily reacts with the silanol groups, combining siloxane chains with the separation of acetic acid. This occurs in according to the general reaction equation presented in Equation (1):

(1) $$∼OSi\left(R_{2}\right)OH+CH_{3}Si{\left(OCOCH_{3}\right)}_{3}+2HOSi\left(R_{2}\right)O∼\overset{-CH_{3}COOH}{\to }∼OSi\left(R_{2}\right)OSiCH_{3}\left[OSi\left(R_{2}\right)O∼\right]$$

Colloidal silicone was obtained by the peptization process using a widely available universal silicone (Soudal N.V., Turnhout, Belgium), which general characteristics is given in Table 4.

As a continuous phase, the tetrafluoroethylene (TFE), was used. Concentration of dispersed phase was 25 wt%, it provided the consistency which dispersion was able to penetrate the intergranular spaces of treated grinding wheel. After evaporation of the continuous phase, self-acting crosslinking of the silicone have occurred. The variation of dispersed phase concentration enables adjustment of the grinding wheel’s quantity of impregnating substance and/or structure to own needs.

**Figure 2:**
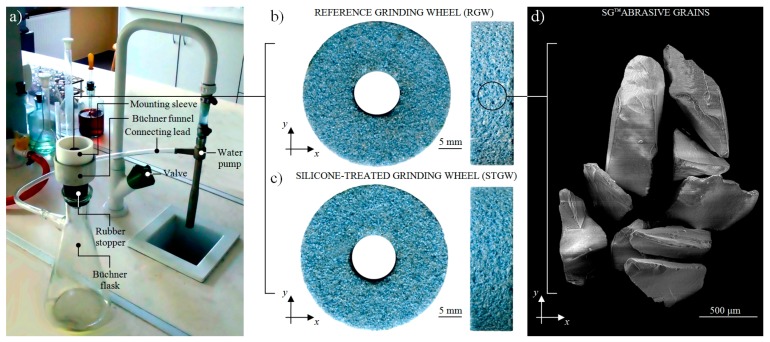
(**a**) Impregnation process of small-size grinding wheel 1-35×10×10SG/F46G10VTO by synthetic organosilicon polymer-based impregnate (silicone) under the reduced pressure conditions: general view of stand for realizing the impregnation process; (**b)** general view of the non-treated RGW; (**c**) STGW after the impregnation process; (**d**) SEM micrograph presenting morphology of SG^™^ abrasive grains (mag.: 50×, Ua = 20 kV, WD = 47 mm, spot size: 39, vacuum pressure: 8 Pa) [58].

The impregnation process was carried out in a special prepared stand, of which the general view is presented in Figure 2a. The grinding wheel was mounted in the mounting sleeve, which was fixed on the upper part of Büchner’s funnel coupled by rubber stopper with a Büchner’s flask and water pump. The impregnating substance saturation of the abrasive tool under reduced pressure was carried out after pouring of its small amount on the grinding wheel surface. The general view of the RGW and STGW was presented in Figure 2b,c. The visual observation of both grinding wheels does not reveal significant differences due to the transparency of the impregnating substance. Additionally, in Figure 2d, an SEM micrograph presenting the morphology of SG™ abrasive grains [58], forming a structure of above abrasive tools, was shown. The measured mass of the abrasive tool before the beginning of the process was *m*_1_ = 17.33 g, whereas the mass after the process increased by 4.15% to values of *m*_2_ = 18.05 g. Measurements were carried out using PS 2100.R2 precision balance produced by Radwag (Radom, Poland).

### 3.2. CLSM-based Analysis of Correctness of Introduction the Impregnating Substance into the GWAS

Proper verification of the correctness of introduction the impregnating substance into the GWAS structure is a complex task, which requires not only mass measurements of grinding wheels before and after the impregnation process, but also the use of more advanced methods of assessment. For this purpose, the authors used one of the modern varieties of confocal microscopy, confocal laser scanning microscopy (CLSM), which provide a high accuracy of obtained results in such application.

**Figure 3:**
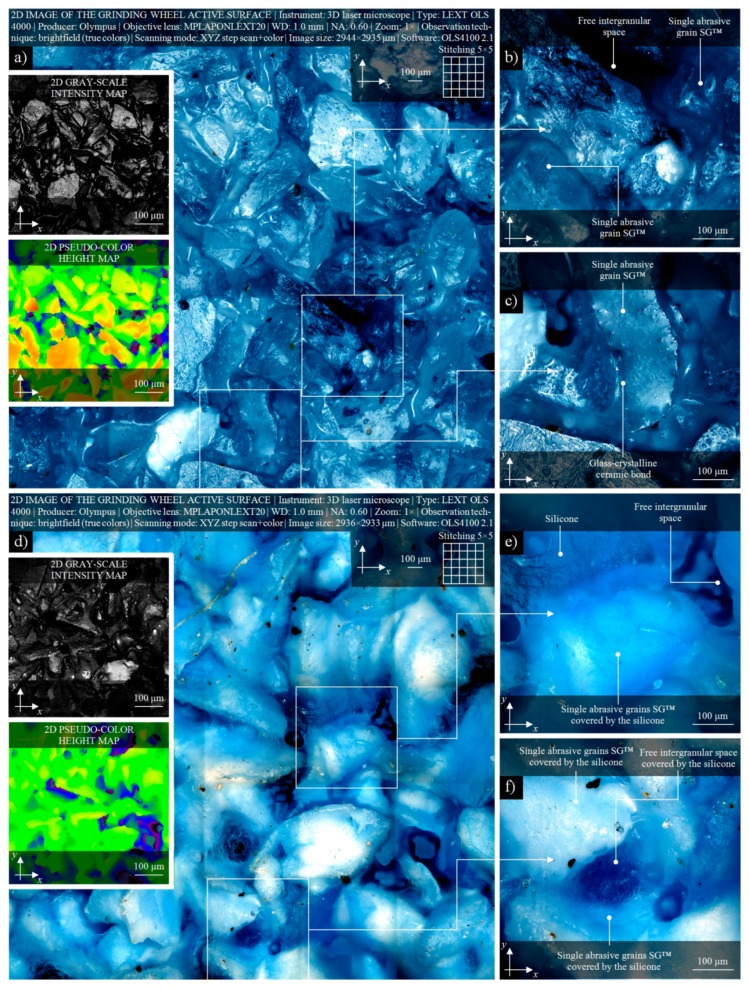
(**a**) Observation and visual analysis of correctness of introduction of the synthetic organosilicon polymer-based impregnate (silicone) into the active surface of small-size grinding wheel 1-35×10×10 SG/F46G10VTO carried out by the use of 3D laser microscope Olympus LEXT OLS4000: active surface of non-treated RGW-1 (size: 2944 × 2935 μm) with 2D gray-scale intensity map and 2D pseudo-color height map (miniatures, left side top); (**b**,**c**) detailed views of active surface of non-treated RGW-1, each extracted AOIs (size: 0.58 × 0.58 µm); (**d**) active surface of STGW-3 after the impregnation process (size: 2936 × 2933 μm) with 2D gray-scale intensity map and 2D pseudo-color height map (miniatures, left side bottom); (**e**,**f**) detailed views of active surface of STGW-3 after the impregnation process, each extracted AOIs (size: 0.58 × 0.58 µm).

In Figure 3, the selected results of observations and measurements of active surface of the small- size grinding wheel 1-35×10×10SG/F46G10VTO before and after the impregnation process, carried out using 3D laser microscope LEXT OLS4000 (Olympus Corp., Shinjuku, Tokyo, Japan), are presented. From a wide area of active surface of the RGW-1, a relatively large (size: 2944 × 2935 μm) area of interest (AOI) created as a 5 × 5 matrix using an image stitching procedure, was selected for observations. A view of the non-impregnated surface of the RGW-1 in brightfield mode is presented in Figure 3a (size: 4724 × 4720 pixels), whereas two miniatures at its left side showing the confocal images visualized by the use of OLS4100 2.1 software (Olympus Corp., Shinjuku, Tokyo, Japan) as a 2D gray-scale intensity map and 2D pseudo-color height map, respectively. For visual analysis of active surface details of the RGW-1, from Figure 3a extracted two AOIs (size: 0.58 × 0.58 µm), presented in Figure 3b,c, respectively. This set obtained for RGW-1, including brightfield image and confocal maps, was extremely helpful during observation and analysis of surface condition of the abrasive tool before the impregnation process. Allowed to observe a very open structure of the small- size grinding wheel 1-35×10×10SG/F46G10VTO with its characteristic elements, e.g., the morphology of a single SG^™^ abrasive grains, areas with glass-crystalline ceramic bonds and free intergranular spaces.

The condition of active surface after the impregnation process is presented in Figure 3d. A similar area (size: 2936 × 2933 μm) of the STGW-3 was measured by 3D laser microscope LEXT OLS4000 (Olympus Corp., Shinjuku, Tokyo, Japan). From input image (size: 4711 × 4708 pixels), a 2D gray-scale intensity map and 2D pseudo-color height map were obtained (miniatures, left side bottom). Additionally, as in previous set, two AOIs (size: 0.58 × 0.58 µm) with detailed view of active surface, were extracted. In these images, presented in Figure 3e,f, a single SG^™^ abrasive grains coated with a thin layer of silicone as well as free intergranular spaces filled with it, which indicates a relatively high level of impregnation process of the abrasive tool, are presented. The carried out visual analysis showed that the impregnating substance was correctly introduced into the volume of the abrasive tool, significantly reducing the openness of its structure and volume of free intergranular spaces as well as effective reducing (approx. two times) the heights of surface irregularities.

### 3.3. EDXRF-Based Microanalysis of Elemental Distribution of the GWAS after the Impregnation Process 

**Figure 4:**
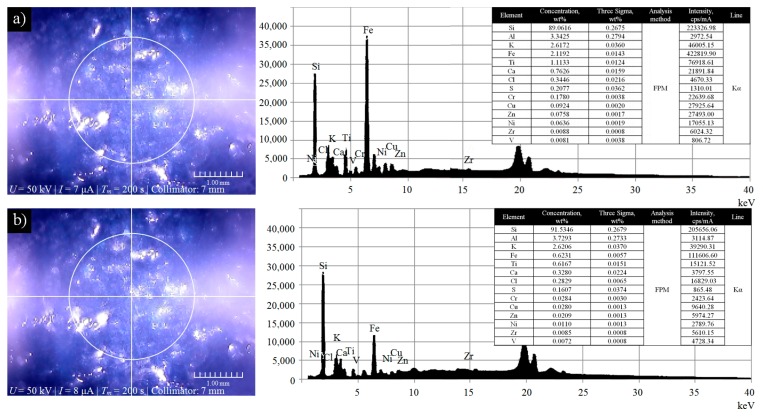
Elemental microanalysis of correctness of introduction of the synthetic organosilicon polymer-based impregnate (silicone) into the active surface of small-size grinding wheel 1-35×10×10 SG/F46G10VTO carried out by the use of X-ray fluorescence analyzer Horiba Mesa 50: (**a**) imaging of analyzed area (Area 1) of the SGTW-2 (**left**), spectrogram and obtained results of elemental microanalysis (**right**); (**b**) imaging of analyzed area (Area 2) of the SGTW-2 (**left**), spectrogram and obtained results of elemental microanalysis (**right**).

Other method used by the authors for verification of the correctness of introduction the impregnating substance into the GWAS structure was energy dispersive X-ray fluorescence (EDXRF). These advanced X-ray fluorescence techniques were used for elemental microanalysis of active surface of the STGW after the impregnation process. The measurements were carried out using X-ray fluorescence analyzer Mesa 50 produced by Horiba, Ltd. (Kyoto, Japan) in ten randomly selected areas on the active surface of the STGW-2. Examples of two microanalyses of selected areas (Area 1 and 2) located in a close distance to each other are presented Figure 4.

Analyzing the obtained spectrograms and results of elemental microanalysis (given in the tables) can be concluded that the concentration of the Si in both cases is highest from the given group of elements (Area 1, *Si* = 89.06 wt.% at *I_f_* = 223,326.98 cps/mA, Area 2, *Si* = 91.53 wt.% at *I_f_* = 205,656.06 cps/mA, respectively). Silicon is one of the main ingredients of colloidal synthetic organosilicon polymer (silicone) used as an impregnating substance. A high concentration of this element indicates that the impregnation process was carried out correctly and the impregnating substance was completely introduced into the GWAS structure. Results obtained by the EDXRF in combination with the results from CLSM (Section 3.2) confirm both the correctness of the methodology adopted and the effectiveness of the used impregnation process.

## 4. Internal Cylindrical Grinding of INCONEL^®^ Alloy 600 by STGW 

### 4.1. Methodology of Experimental Studies

The goal of experimental studies was to determine the influence of impregnation by silicone of the grinding wheels on the course and effects of the internal cylindrical grinding process used for machining of internal surfaces of the rings made of INCONEL^®^ alloy 600. The obtained results of carried out experiments using the STGW were referred to the results obtained by RGW. Using both types of the grinding wheels, the same volume of material (*V_w_* = 3142 mm^3^) was removed during grinding, corresponding to the machining of ten internal surfaces of the rings at ground material removal rate (*Q_w_* = 5.24 mm^3^/s). The grinding process conditions are collectively presented in Table 5.

During experimental studies, the data regarding with grinding power, volume of material removed, and surface microtopography parameters was collected. In relation to workpiece surface, the measurements were carried out using stylus profilometer Hommel–Tester T8000 produced by Hommelwerke GmbH (Villingen-Schwenningen, Germany), whereas 3D laser microscope LEXT OLS4000 (Olympus Corp., Shinjuku, Tokyo, Japan) was used for measurements of active surface of the non-impregnated RGW and STGW after the impregnation process. Additionally, for determining the possible influence of silicone impregnation on the temperature of the grinding wheel and the workpiece, thermograms of the active surface were also acquired using IRT camera Testo 890 produced by Testo SE & Co. KGaA (Titisee-Neustadt, Germany).

### 4.2. Stylus Profilomery and CLSM-Based Analysis of the Surface Microgeometry

**Figure 5:**
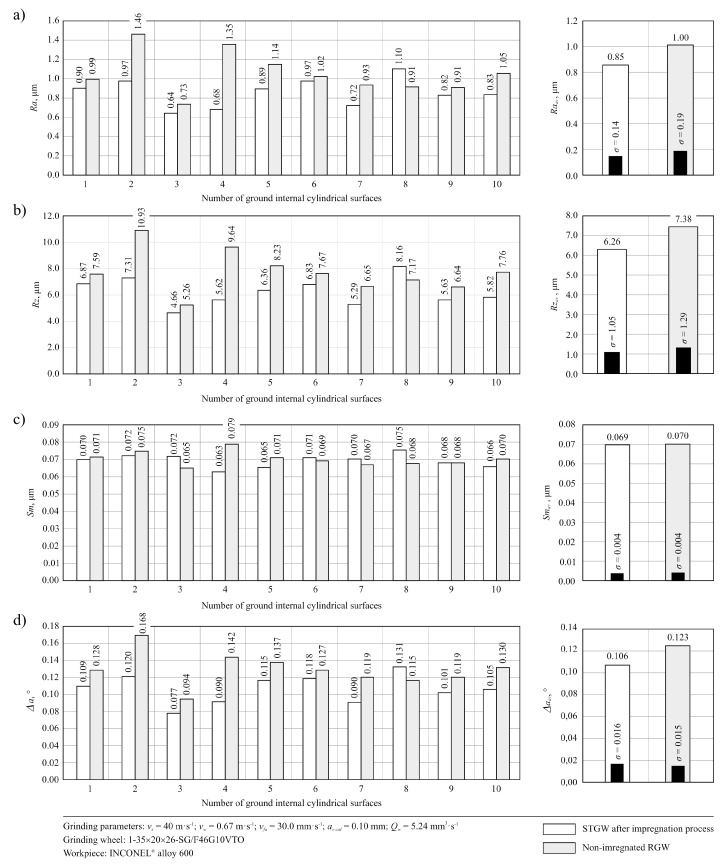
Bar charts presenting changes in values of selected set of parameters obtained during the measurements of ground surface microgeometry machined by STGW and RGW carried out using stylus profilometer Hommelwerke Hommel–Tester T8000: (**a**) *Ra*; (**b**) *Rz*; (**c**) *Sm*; (**d**) *Δa*.

**Figure 6:**
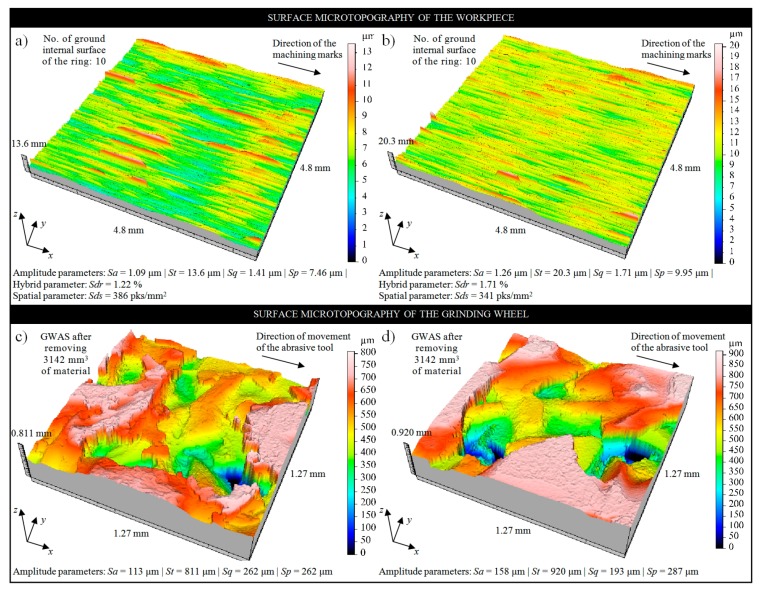
Collection of example results of measurements obtained after the grinding process of the rings made of INCONEL^®^ alloy 600 using 3D laser microscope Olympus LEXT OLS4000: microtopographies of internal surface of the ring No. 10 machined by: (**a**) STGW; (**b**) RGW; microtopographies of the active surface of: (**c**) STGW; (**d**) RGW; after ground of ten workpieces.

In Figure 5, the changes in values of selected set of parameters (*Ra*, *Rz*, *Sm* and *Δa*) obtained during the measurements of ground surface microgeometry using STGW and RGW, are presented. Comparison of the results of carried out measurements shows that approx. 15% lower values of roughness parameters of the ground surface were registered for STGW. This trend was also confirmed by the analysis of microtopographies of the machined surfaces obtained using 3D laser microscope Olympus LEXT OLS4000. A good example is, in this case, microtopographies obtained for internal surface of the ring No. 10, which was effectively machined by STGW and RGW (Figure 6a,b). The obtained results of the measurements suggest that the introduction of impregnating substance in a form of synthetic organosilicon polymer-based impregnate (silicone)—which was effectively introduced to the volume of the grinding wheel as shown in Figure 3 and Figure 4—had a positive effect on the roughness of the machined surface. This influence seems to be a relatively small but noticeable throughout the entire durability period of the grinding wheel.

For determining the causes of such influence of the impregnating substance on the grinding process, the detailed analyses of condition of the active surface of both types of grinding wheels after the process stopped were carried out. Several microscopic observations and measurements of the GWAS microtopography were carried out, examples of which are given in Figure 6c,d. Detailed analysis of the STGW has shown that after grinding process on its active surface, impregnating substance cannot be observed (Figure 6c)—which is in contrast to the state of the grinding wheel after impregnation (before machining) shown in Figure 3. Compared to the surface of the RGW (Figure 6d), only a smaller distribution of microcloggings located on vertexes of the abrasive grains can be seen.

To explain the lack of silicone on the GWAS after work (Figure 6c) the combustion process of pure silicone was carried out. In this case, three test portions of different masses were initially combusted in a ceramic crucible on a gas burner to allow complete combustion, and then the residue after combusting was calcined in a muffle furnace for about two hours until a constant weight of the crucible was obtained. The average weight loss of silicone was about 70%. As a result of combustion, a white solid was formed. This material was subjected to qualitative chemical analysis, i.e., the solubility in hydrochloric acid, sulfuric acid VI (both concentrated and diluted) and in hydrofluoric acid was analyzed. This substance was dissolved only in hydrofluoric acid.

Based on the carried out chemical analysis, it can be assumed that in high temperature conditions, which occurred in the GZ, the silicone introduced into the pores of the grinding wheel has decomposed due to its limited heat resistance (about 200 °C, 392 °F).

As a result of silicone decomposition, the silica (SiO_2_), was created. This high hardness (about 7.0 on the Mohs scale) compound contributed to effective smoothing the vertices of the workpiece surface irregularities. Silica, in this case, can be treated as a kind of loose abrasive, which is near the GZ. This means that the impregnating substance in the form of silicone, originally introduced into the volume of the grinding wheel to obtain the effect of reducing the chip adhesion forces of the ground workpiece to the GWAS, as a result, has significantly contributed to reducing the height of irregularities of the machined surface.

**Figure 7:**
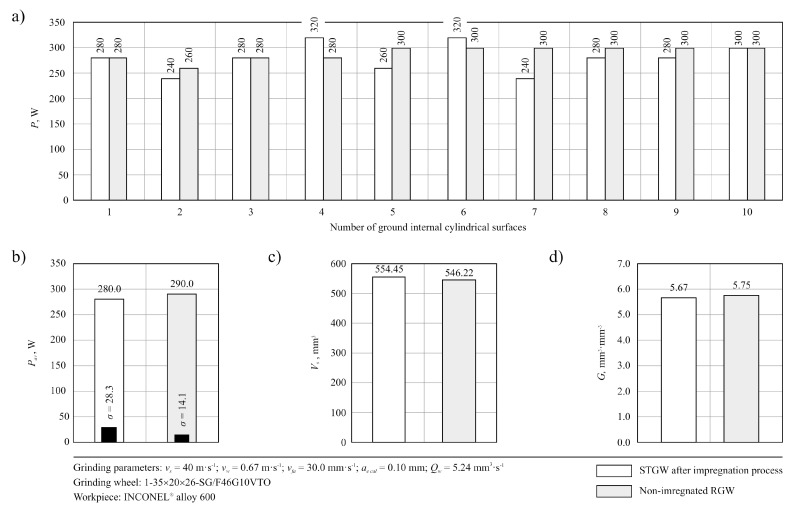
Collection of exemplary results, including values of grinding power and values of parameters describing the durability period of the grinding wheels studied: (**a**) changes in grinding power *P* during machining of internal surfaces of 10 rings; (**b**) average value of the grinding power *P_avr_* and its standard deviation *σ*; (**c**) volumetric wear of the grinding wheel *V_s_*; (**d**) grinding ratio *G* = *V_w_*/*V_s_*.

This conclusion seems to be confirmed by the values of the *Sp* amplitude (surface) parameter determined based on the obtained microtopography measured for internal surface of the ring No. 10 machined two types of grinding wheels, presented in Figure 6a,b. The value of maximal peak height for surface machined by the STGW was *Sp* = 7.46 μm (Figure 6a), whereas for surface machined by the RGW was about 30% higher—*Sp* = 9.96 μm (Figure 6b). Much lower differences between the compared grinding wheels were obtained in the case of grinding power *P*, which is presented in Figure 7a. Other results, including values of volumetric wear of the grinding wheel *V_s_* and grinding ratio *G* = *V_w_*/*V_s_* are presented in Figure 7c,d, respectively. The relatively high volumetric wear *Vs* of both grinding wheels results from their very open structure (No. 10), which was selected due to the possibility of penetration of the impregnating substance deep into the abrasive tool, which did not ensure sufficiently strong bonding of abrasive grains in the examined process. For structure No. 10 and hardness G, the volume of bond in the grinding wheel is 11.5%, at 42% of grains volume and 46.5% of pores. Despite the use of a special glass-crystalline ceramic bond, with approx. 40% of the spinel phase gahnite and willemite, the resistance of grinding wheels to volumetric wear proved to be insufficient. Obtained relatively low values of the grinding ratio *G* (Figure 7d) also indicate this effect.

### 4.3. IRT-Based Analysis of Influence of Impregnation Process on Grinding Process Temperature 

**Figure 8:**
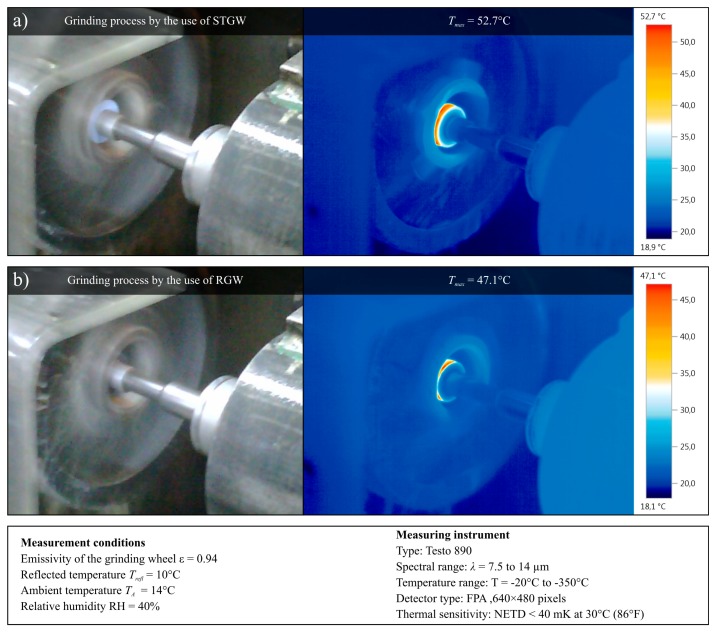
Collection of example results of thermal analysis presenting the temperature (thermograms, right side) during the grinding process (view of the GZ, left side) realized with: (**a**) STGW; (**b**) RGW, acquired using IRT camera Testo 890.

To determine the possible influence of impregnation of the grinding wheels by the synthetic organosilicon polymer-based impregnate (silicone) grinding wheel impregnation on the grinding process temperature, the thermograms of the working area were acquired during machining with the both grinding wheels. In Figure 8, examples of thermograms showing the temperature during the grinding process using a STGW (Figure 8a) and RGW (Figure 8b) are presented. Despite these limitations, a comparative analysis of over two hundred thermograms acquired during the grinding process realized with STGW and RGW was carried out. As a result, it was found that the introduction of an impregnating substance in the pores of the grinding wheel does not affect the grinding temperature in a significant way, whose changes can be observed and acquired with the measuring equipment (IRT camera Testo 890, Testo SE & Co. KGaA, Titisee-Neustadt, Germany) used.

The presented results of carried out experimental studies showed a beneficial effect of the introduction of impregnating substance into the grinding wheel volume on reducing the high of irregularities of the machined surface. One of the possible phenomena causing this effect is the thermal decomposition of silicone during the grinding process with the release of high hardness silicon dioxide, which can act as a loose abrasive, which effectively smoothing the ground surface.

## 5. Conclusions 

The carried out experimental studies of internal cylindrical grinding process of workpieces made of INCONEL^®^ alloy 600 by STGW allowed the formulation of the following conclusions:

- The need for effective, fast and relatively cheaper introduction of anti-adhesive and lubricating substances into the free intergranular spaces of ceramic bonded grinding wheels has led to the development of several innovative impregnation methods used as an impregnate many type of a non-toxic substances (Section 1).
- One of them is developed by the authors method of directly introducing an impregnating substance into the structure of the abrasive tool (Section 3.1). This method is characterized by the possibility of adjusting the amount of impregnating substance introduced into the grinding wheel (which is extremely important from the point of view of maintaining the ability of the GWAS to transport GF to the GZ and receiving grinding process products from it) and relatively low costs of implementation into industrial practice.
- The impregnation process realized with the use of synthetic organosilicon polymer-based impregnate (silicone) (Section 3.1) was intended to have a positive effect on the course and results of the grinding process of nickel alloy, in particular it was expected to achieve the effect of reducing the adhesion between the GWAS and grinding process products, including mainly chips of the workpiece.
- Obtained results of the experimental studies incline to formulate the hypothesis about the possibility of a beneficial effect of impregnating substance on reducing the height of irregularities of the machined surface (Section 4.2 and Section 4.3), resulting from the thermal decomposition of this compound during the grinding process with the release of high hardness silicon dioxide, which as a loose abrasive can effectively smoothen the ground surface.
- Presented results of experimental studies can be treated as a basis for developing guidelines for the selection of the grinding wheel structure and the selection of grinding conditions for nickel superalloys to increase the use of the potential of STGWs.
- In further works, a more comprehensive analysis of the material resulting from the decomposition of silicone to determine the variety of silica and its microstructure, is necessary. Advanced electron microscopy methods (SEM) and various variations of methods using X-ray spectroscopy (EDS, EDXRF) can be extremely helpful in this case.

## Figures and Tables

**Table 1:** Review of selected substances used for the impregnating process of grinding wheels.

Impregnating Substance		References			
Group	Name	Year	Patent	Year	Paper
Non-metallic elements and their derivatives	Sulfur	1927	Harmann [11]	1983	Sakuma and Tado [12]
		1941	Jones [13]	1985	Younis and Alawi [14]
		1943	Jackson [15]	2003	Salmon [16]
		1951	Coes [17]	2004	Holesovsky and Hrala [18]
		1967	Gallagher [19]	2013	Rudometov [20]
		2002	Krueger et al. [21]	2015	Nadolny et al. [22]
	Graphite	1967	Hunsberger and Tucker [23]	2003	Shaji and Radhakrishnan [24]
				2005	Irani et al. [25]
		1972	Sioui and Cohen [26]	2009	Alberts et al. [27]
				2012	Tsai and Jian [28]
				2013	Rudometov [20]
	Amorphous carbon	2011	Sienicki et al. [29]	2015	Nadolny et al. [30,31]
Metallic elements	Bismuth alloy	1970	Meyer [32]	–	–
Organic chemical compounds	Wax	1980	Kunimasa [33]	1969	Weeks and Osborne [34]
	Wax + Oil	1999–2004	Rossetti Jr. et al. [35,36,37]		
	(Wax) + Paraffin	1969	Ackermann Jr. et al. [38]	1971	Svekrov [39]
				2007	Chirkov [9]
Inorganic chemical compounds	Hexagonal boron nitride	1970	Mathewson and Owens [40]	2017	Wojtewicz [41]
Solids nanoparticles	Molybdenum disulfide	1995	Serdyuk et al. [42]	2016	Zhang et al. [43]
		2010	Hashimoto and Iketani [44]	2017	Wojtewicz [41]
		2012	Bo et al. [45]	2019	Kapłonek et al. [46]
		2015	Zhiqi et al. [47]		
	Graphene	–	–	2016	Ravuri et al. [48]
				2019	Paven et al. [49]
	Carbon nano-tubes	–	–	2015	Li et al. [50]
Organosilicon compounds	Silicone	2001	Maeda et al. [51]	2019	Kapłonek et al. [52]
				2019	Nadolny et al. [53]
Polymer compounds	Epoxy resin	1939	Hudson [54]	1994	Mulla and Krstic [55]
		1970	Amero [56]		

**Table 2:** General characteristics of the grinding wheels used in the experimental studies.

Technical Designation	1-35×10×10-SG/F46G10VTO		
Producer	Subject Group of Fundamental of Materials Science and Technical Ceramics, Faculty of Technology and Education, Koszalin University of Technology, Koszalin, Poland		
Grinding wheel type	1—flat grinding wheel		
Dimensions	*d_s_* = 35 mm, *b_s_* = 10 mm, *h_s_* = 10 mm		
Abrasive grain type	Microcrystalline sintered corundum SG™ (Norton, Worcester, MA, USA)		
Abrasive grain fracture No.	46		
Hardness class	G		
Structure No.	10		
Bond	Special vitrified (V) bond with glass-crystalline microstructure		
Volume of grains (*V_g_*)	42.0%		
Volume of bond (*V_b_*)	11.5%		
Volume of pores (*V_p_*)	46.5%		
Experimental studies	**Designation**	**Surface Condition**	**Pieces**
	RGW-x (Reference grinding wheel)	Non-impregnated	2
	STGW-x (Silicone-treated grinding wheel)	Impregnated	8

x—Individual number of the grinding wheel, e.g., RTG-1, STGW-5.

**Table 3:** Chemical composition of INCONEL^®^ alloy 600 ^a^, as well as its selected physical, mechanical, and thermal properties.

Element	Concentration (%)	Physical Properties			
Ni + Co	72.00 min	Parameter		Value	Unit
Cr	14.00–17.00	Density		8.47	g/cm^3^
Fe	6.00–10.00	Melting range		1354–1413 ^b^	°C
C	0.15 max.	Modulus of	rigidity	75.6	kN/mm^2^
Mn	1.00 max.		elasticity	206	kN/mm^2^
Si	0.50 max.	**Mechanical Properties**			
S	0.015 max.	Elongation at break		45	%
Cu	0.50 max.	Hardness (Brinell)		≤185	kg/mm^2^
**Material No.**		Yield strength		340	MPa
2.4816		Tensile strength		550	MPa
**Standard**	**Common Trade Name**	**Thermal Properties**			
UNS N06600	INCONEL^®^ alloy 600 (Special Metals Corp.)	Coefficient of thermal expansion		11.5–13.3 ^c^	μm/m
ASTM B167		Thermal conductivity		14.8–15.9 ^d^	W/m·°C
	ATI 600™ (Allegheny Technologies Inc.)	Curie temperature		−194 ^e^	°C
ASME SB167		Specific heat		444 ^d^	J/kg·°C

^a^ Used in the experimental studies. Alloy was produced by Special Metals Corp. (New Hartford, NJ, USA) and distributed by Bibus Metals AG (Fehraltorf, Switzerland); ^b^ 2470–2575 °F, respectively; ^c^ for temperatures in a range from 20–100 °C (70–212 °F); ^d^ for temperature 20 °C; ^e^ −317 °F.

**Table 4:** General characteristics of universal silicone used during impregnation process.

Property	Test Method	Feature/Value
Basis		Polysiloxane
Consistency		Stable paste
Curing system		Polymerization with involving of moisture
Type of curing		Acid (acetoxy)
Skin formation	at 20 °C (68 °F), 65% RH ^a^	~20 min
Curing speed	at 20 °C (68 °F), 65% RH ^a^	~2 mm/24 h
Hardness	Shore A	20 ± 5
Specific weight		0.95 g/cm^3^
Density		~1.03 g/mL
Maximum allowed distortion		25%
Max. tension	DIN 53504 [57]	1.35 N/mm²
Elasticity modulus	DIN 53504 [57]	0.23 N/mm²
Elongation at break	DIN 53504 [57]	800%
Application temperature		From 5 °C (41 °F) to 35 °C (95 °F)

^a^ At high temperatures, the machining and curing times in the cross section are shortened, in the low temperatures, the times are longer.

**Table 5:** Characteristics of grinding process conditions.

**Grinding process**	Variety: Reciprocal peripheral internal cylindrical grinding
**Grinding machine**	Universal grinding machine: RUP 28P produced by Tarnów Mechanical Works S.A. (Tarnów, Poland)
**Grinding wheel**	Grinding wheel: Small-sized sol–gel alumina 1-35×10×10-SG/F46G10VTO Number of pieces: 2 (non-impregnated RGWs), 8 (STGWs after impregnation process)
**Grinding wheel dressing parameters**	Dresser: single grain diamond dresser with mass: *Qd* = 1.25 kt, Grinding wheel rotational speed while dressing: *n_sd_* = 12,000 min^−1^ Dressing allowance: *a_d_* = 0.0125 mm Axial table feed speed while dressing: *v_fd_* = 10 mm·s^−1^ Number of dressing passes: *i_d_* = 12
**Grinding process parameters**	Grinding wheel peripheral speed: *v_s_* = 40 m·s^−1^ Axial table feed speed: *v_fa_* = 30 m·s^−1^ Working engagement (machining allowance): *a_e_* = 0.0075 mm Total working engagement (machining allowance): *a_e tot_* = 0.10 mm Workpiece peripheral speed: *v_w_* = 0.67 m·s^−1^ Total grinding time *t_g tot_* = 80 s
**Grinding fluid**	Grinding fluid: 5% water solution of Syntilo RHS oil produced by Castrol Ltd. (Liverpool, Great Britain), delivered using flood method Grinding fluid flow rate: *Q_GF_* = 4.70 L·min^−1^
**Workpiece**	Form of the sample: ring (internal diameter: *d_w_* = 45 mm, width: *b_w_* = 20 mm) Machined surface: internal cylindrical surface of ring Material: INCONEL^®^ alloy 600 Number of pieces: 10

## Nomenclature

AOI	Area of interest
BES	Backscattered electron mode
CLSM	Confocal laser scanning microscopy
EDS	Energy dispersive X-ray spectroscopy
EDXRF	Energy dispersive X-ray fluorescence
FPA	Focal-plane array
FPM	Fundamental parameters method
GF	Grinding fluid
GWAS	Grinding wheel active surface
GZ	Grinding zone
IRT	Infrared thermography
RGW	Reference grinding wheel (non-treated)
NA	Numerical aperture
NETD	Noise equivalent temperature difference
SEM	Scanning Electron Microscopy
SG^™^	Trade name of submicrocrystalline alumina abrasive grains produced by Saint-Gobain Abrasives (Courbevoie, France) using Seeded Gel technology
STGW	Silicone-treated grinding wheel
TFE	Tetrafluoroethylene
WD	Working distance, mm
*a_d_*	Dressing allowance, mm
*a_e_*	Working engagement (machining allowance), mm
*a_e tot_*	Total working engagement (machining allowance), mm
*b_s_*	Width (grinding wheel), mm
*b_w_*	Width (workpiece), mm
*d_s_*	External diameter (grinding wheel), mm
*d_w_*	Internal diameter (workpiece), mm
*h_s_*	Internal diameter (grinding wheel), mm
*i_d_*	Number of dressing passes, –
*m*_1_	Mass of the grinding wheel before impregnation process, g
*m*_2_	Mass of the grinding wheel after impregnation process, g
*n_sd_*	Grinding wheel rotational speed while dressing, min^−1^
*t_g tot_*	Total grinding time, s
*v_fa_*	Axial table feed speed, mm·s^−1^
*v_fd_*	Axial table feed speed while dressing, mm·s^−1^
*v_s_*	Grinding wheel peripheral speed, m·s^−1^
*v_w_*	Workpiece peripheral speed, m·s^−1^
*C*	Concentration, wt.%
*G*	Grinding ratio, mm^3^/mm^3^
*I*	Current, μA
*I_f_*	Fluorescence intensity, cps/mA
*P*	Grinding power, W
*Q_d_*	Diamond dresser mass, kt
*Q_GF_*	Grinding fluid flow rate, L·min^−1^
*Q_w_*	Material removal rate, mm^3^/s
*Ra*	Arithmetical mean deviation of the roughness profile, μm
*Rz*	Maximum height of the profile within a sampling length, μm
*Sa*	Arithmetic mean deviation of the surface, μm
*Sdr*	Developed interfacial area ratio, %
*Sds*	Density of summits of the surface, pks/mm^2^
*Sm*	Mean spacing of profile irregularities, μm
*Sp*	Maximum peak height, µm
*Sq*	Root mean square deviation of the surface, µm
*St*	Total height of the surface, μm
*T_m_*	Measurement time, s
*U*	Voltage, kV
*V_b_*	Volume of bond, %
*V_g_*	Volume of grains, %
*V_p_*	Volume of pores, %
*V_s_*	Volumetric wear of the grinding wheel, mm^3^
*V_w_*	Volume of material removed, mm^3^
*σ*	Standard deviation
*Δa*	Average absolute slope, °
